# Toward a Meta-Analytic Synthesis of the Resting-State fMRI Literature for Clinical Populations

**DOI:** 10.1155/2015/435265

**Published:** 2015-06-10

**Authors:** Yu-Feng Zang, Xi-Nian Zuo, Michael Milham, Mark Hallett

**Affiliations:** ^1^Center for Cognition and Brain Disorders, Hangzhou Normal University, Hangzhou 310015, China; ^2^Zhejiang Key Laboratory for Research in Assessment of Cognitive Impairments, Hangzhou Normal University, Hangzhou 310015, China; ^3^Key Laboratory of Behavioral Science and Magnetic Resonance Imaging Research Center, Institute of Psychology, Chinese Academy of Sciences, Beijing 100101, China; ^4^Center for the Developing Brain, Child Mind Institute, New York, NY 10022, USA; ^5^Center for Biomedical Imaging and Neuromodulation, Nathan S. Kline Institute for Psychiatric Research, Orangeburg, NY 10962, USA; ^6^Human Motor Control Section, National Institute of Neurological Disorders and Stroke, NIH, Bethesda, MD 20892, USA

Coordinate-based meta-analysis (CB-meta) is playing an important role in identifying spatially consistent findings for targeted questions in the neuroimaging literature, by quantitatively aggregating independent results reported in a standard coordinate space [1]. CB-meta has been widely used in voxel-based magnetic resonance imaging (MRI) studies, including task functional MRI (fMRI), where either patterns of within-group activation or patterns of between-group activation differences can be aggregated across studies. Given that variations in task design can introduce confounds into such pursuits, researchers work to limit any effort to the inclusion of studies using highly similar tasks. Task fMRI CB-meta papers have been published in most brain disorders.

Resting-state fMRI (RS-fMRI) does not require cognitive task probes, and its design is inherently similar across studies. From this perspective, it is ideally suited for CB-meta. However, while an array of analytic methods have emerged to characterize different aspects of resting brain activity, not all of them are suitable for CB-meta. This likely explains the relatively small number of RS-fMRI based CB-meta published to date [2–8].

The analytic methods for RS-fMRI can be divided into two categories, one for depicting functional relationships between remote brain regions and another for local activity (for a systematic review, see [9]). The widely used methods in the former category include seed-based functional connectivity, spatial independent component analysis (sICA), and graph theory. The latter has two widely used methods, namely, regional homogeneity (ReHo) and amplitude of low frequency fluctuation (ALFF)/fractional ALFF (fALFF).

Seed-based functional connectivity is one of the most widely used analytic methods in RS-fMRI studies. Typically, a region of interest (ROI) is predefined and then linear correlation or linear regression analysis is performed between the mean time series of this ROI and the time series of each voxel in the brain. The results are of course dependent on the location of seed ROIs and therefore are not suitable for CB-meta. Most of the 8 CB-meta RS-fMRI papers aforementioned did not include seed-based functional connectivity studies.

Spatial independent component analysis (sICA) decomposes the RS-fMRI data into multiple networks (components), among which only about 10 networks are psychophysiologically interpretable [10]. “Spatial independent” means spatially nonoverlapping. Therefore, sICA papers should not be taken into a CB-meta study, unless different papers have focused on the same component (e.g., the default mode network). A few CB-meta RS-fMRI studies have included sICA RS-fMRI papers [2, 4, 6, 8]. However, few explicitly mentioned the limitation of sICA for CB-meta.

Graph theory has been widely used for exploring the topological organization of complex brain networks in RS-fMRI studies. Unlike seed-based functional connectivity and sICA, which focus on a specific functional system(s), graph theory usually explores the topological properties, such as small-world, modular structure and highly connected hubs, of the entire brain. From this point of view, RS-fMRI studies using graph theory are also well suitable for meta-analysis. However, due to the high computational cost, most graph-based RS-fMRI studies have chosen a limited number of brain regions or ROIs, rather than brain voxels, as network nodes. Such results do not provide the coordinate information needed to support CB-meta. Degree centrality is one of the simplest and least computation-demanding measures for graph theory complex networks. Several RS-fMRI toolboxes (e.g., DPARSF (http://www.restfmri.net/; http://rfmri.org/) and Gretna (https://www.nitrc.org/projects/gretna/)) have included voxel-based degree centrality measurement [11, 12]. Some studies have applied voxel-based centrality to brain disorders including depression [13–15] and Alzheimer's disease [16]. In light of recent increases in computational capacity, more complicated measurements of graph theory will be implemented in a voxel-wise manner, thereby increasing the suitability of graph theory for CB-meta.

ReHo and ALFF are two methods widely used for characterizing local spontaneous activity of RS-fMRI data. ReHo measures the local synchronization of the time series of neighboring voxels [17] whereas ALFF/fALFF measures the amplitude of time series fluctuations at each voxel [18, 19]. Although both ReHo and ALFF/fALFF measure the local activity of each voxel, many studies used the two measurements and suggested that the two methods reveal different aspects of brain function and abnormalities arising in clinical populations [20–23]. For nearly all of the existing CB-meta RS-fMRI studies, researchers have combined across these methods for characterizing local activity (ReHo and/or ALFF), despite known differences in their properties. A CB-meta RS-fMRI study of depression by Iwabuchi and colleagues for the first time included RS-fMRI papers using the same analytic method (ReHo) [5]. This approach markedly reduces discrepancies in analytic methods. However, only 10 of the 200+ RS-fMRI papers on depression to date met the inclusion criteria of that CB-meta study.

Clinical studies always face the challenges of high heterogeneity and limited sample size in patient groups. Therefore, meta-analysis is critical for drawing congruent conclusion across studies carried out with similar settings and techniques. Few techniques for clinical studies enable the application of the broad range of analytic methods that RS-fMRI does in an effort to reveal the functional complexity of the human brain from multiple aspects. Although some analytic methods are not suitable for CB-meta, the strength of RS-fMRI is that its design is inherently similar across studies. Therefore, each dataset could be reanalyzed by using analytic methods being suitable to perform CB-meta. The current special issue was launched to encourage RS-fMRI studies on brain disorders by reanalyzing the published data with methods supporting future CB-meta.

In the current issue, a paper from Dr. Chunshui Yu's group in Tianjin Medical University is of particular interest (see Y. Xu et al., “Altered Spontaneous Brain Activity in Schizophrenia: A Meta-Analysis and a Large-Sample Study”). The authors not only performed two CB-meta RS-fMRI studies, in which only RS-fMRI papers using methods for local activity (ReHo and ALFF, resp.) were included, but they also validated the CB-meta results in their own dataset obtained from a relatively large sample of schizophrenia patients. One of the congruent results was that ALFF was reduced in the primary visual and primary sensorimotor cortex (see details in Y. Xu et al. “Altered Spontaneous Brain Activity in Schizophrenia: A Meta-Analysis and a Large-Sample Study”). The limitation of this study, like other CB-meta RS-fMRI studies, is the small number of eligible RS-fMRI papers. Only 6 ALFF papers and 4 ReHo RS-fMRI papers were included.

We hope this special issue will draw attention to the need for and value of CB-meta in the RS-fMRI research field. If your dataset has not been analyzed with a method that facilitates future CB-meta, please try it. Future CB-meta efforts need large numbers of studies for inclusion to enable more definitive conclusions, upon which models for the prediction or diagnosis of brain disorders can be developed. Additionally, accurate localization of abnormal spontaneous brain activity may help to guide intervention therapies (e.g., deep brain stimulation, transcranial magnetic stimulation, or transcranial ultrasound stimulation).

## References

[1] Eickhoff S. B., Laird A. R., Grefkes C., Wang L. E., Zilles K., Fox P. T. (2009). Coordinate-based activation likelihood estimation meta-analysis of neuroimaging data: a random-effects approach based on empirical estimates of spatial uncertainty. *Human Brain Mapping*.

[2] Kühn S., Gallinat J. (2013). Resting-state brain activity in schizophrenia and major depression: a quantitative meta-analysis. *Schizophrenia Bulletin*.

[3] Graham J., Salimi-Khorshidi G., Hagan C. (2013). Meta-analytic evidence for neuroimaging models of depression: state or trait?. *Journal of Affective Disorders*.

[4] Sundermann B., Beverborg M. L., Pfleiderer B. (2014). Toward literature-based feature selection for diagnostic classification: a meta-analysis of resting-state fMRI in depression. *Frontiers in Human Neuroscience*.

[5] Iwabuchi S. J., Krishnadas R., Li C., Auer D. P., Radua J., Palaniyappan L. (2015). Localized connectivity in depression: a meta-analysis of resting state functional imaging studies. *Neuroscience & Biobehavioral Reviews*.

[6] Palmer S. M., Crewther S. G., Carey L. M., START Project Team (2015). A meta-analysis of changes in brain activity in clinical depression. *Journal of Frontiers in Human Neuroscience*.

[7] Chen Z. Q., Du M. Y., Zhao Y. J. (2015). Voxel-wise meta-analyses of brain blood flow and local synchrony abnormalities in medication-free patients with major depressive disorder. *Journal of Psychiatry & Neuroscience*.

[8] Hannawi Y., Lindquist M. A., Caffo B. S., Sair H. I., Stevens R. D. (2015). Resting brain activity in disorders of consciousness: a systematic review and meta-analysis. *Neurology*.

[9] Zuo X., Xing X. (2014). Test-retest reliabilities of resting-state FMRI measurements in human brain functional connectomics: a systems neuroscience perspective. *Neuroscience & Biobehavioral Reviews*.

[10] Damoiseaux J. S., Rombouts S. A. R. B., Barkhof F. (2006). Consistent resting-state networks across healthy subjects. *Proceedings of the National Academy of Sciences of the United States of America*.

[11] Chao-Gan Y., Yu-Feng Z. (2010). DPARSF: a MATLAB toolbox for ‘pipeline’ data analysis of resting-state fMRI. *Frontiers in System Neuroscience*.

[12] Xu T., Yang Z., Jiang L., Xing X.-X., Zuo X.-N. (2015). A connectome computation system for discovery science of brain. *Science Bulletin*.

[13] Wang L., Dai Z., Peng H. (2014). Overlapping and segregated resting-state functional connectivity in patients with major depressive disorder with and without childhood neglect. *Human Brain Mapping*.

[14] Wang L., Xia M., Li K. (2015). The effects of antidepressant treatment on resting-state functional brain networks in patients with major depressive disorder. *Human Brain Mapping*.

[15] Shen Y., Yao J., Jiang X. (2015). Sub-hubs of baseline functional brain networks are related to early improvement following two-week pharmacological therapy for major depressive disorder. *Human Brain Mapping*.

[16] Dai Z., Yan C., Li K. (2014). Identifying and mapping connectivity patterns of brain network hubs in Alzheimer's disease. *Cerebral Cortex*.

[17] Zang Y., Jiang T., Lu Y., He Y., Tian L. (2004). Regional homogeneity approach to fMRI data analysis. *NeuroImage*.

[18] Zang Y. F., He Y., Zhu C. Z. (2007). Altered baseline brain activity in children with ADHD revealed by resting-state functional MRI. *Brain & Development*.

[19] Zou Q. H., Zhu C. Z., Yang Y. (2008). An improved approach to detection of amplitude of low-frequency fluctuation (ALFF) for resting-state fMRI: fractional ALFF. *Journal of Neuroscience Methods*.

[20] Lei D., Ma J., Du X., Shen G., Tian M., Li G. (2012). Spontaneous brain activity changes in children with primary monosymptomatic nocturnal enuresis: a resting-state fMRI study. *Neurourology and Urodynamics*.

[21] An L., Cao Q.-J., Sui M.-Q. (2013). Local synchronization and amplitude of the fluctuation of spontaneous brain activity in attention-deficit/hyperactivity disorder: a resting-state fMRI study. *Neuroscience Bulletin*.

[22] Premi E., Cauda F., Gasparotti R. (2014). Multimodal FMRI resting-state functional connectivity in *Granulin* mutations: the case of fronto-parietal dementia. *PLoS ONE*.

[23] Han L., Zhaohui L., Fei Y. (2015). Disrupted neural activity in unilateral vascular pulsatile tinnitus patients in the early stage of disease: evidence from resting-state fMRI. *Progress in Neuro-Psychopharmacology and Biological Psychiatry*.

